# Are the Patterns of Cytomegalovirus Viral Load Seen After Solid Organ Transplantation Affected by Circadian Rhythm?

**DOI:** 10.1093/infdis/jiac055

**Published:** 2022-02-20

**Authors:** Hannah Rafferty, Matthew J Murray, Jerry C H Tam, Alastair Macfarlane, Colette Smith, Sheila F Lumley, Sowsan Atabani, Jane A McKeating, Dinesh Sharma, Matthew Reeves, David Whitmore, Paul Griffiths

**Affiliations:** Institute for Immunity and Transplantation, UCL, London, United Kingdom; Institute for Immunity and Transplantation, UCL, London, United Kingdom; Institute for Immunity and Transplantation, UCL, London, United Kingdom; Institute for Immunity and Transplantation, UCL, London, United Kingdom; Institute for Global Health, University College London, London, United Kingdom; Institute for Immunity and Transplantation, UCL, London, United Kingdom; Nuffield Department of Medicine, University of Oxford, Oxford, United Kingdom; Public Health England Birmingham Laboratory, National Infection Service, University Hospitals Birmingham, Bordesley Green East, BirminghamUnited Kingdom; Nuffield Department of Medicine, University of Oxford, Oxford, United Kingdom; Chinese Academy of Medical Sciences, Oxford Institute (COI), University of Oxford, United Kingdom; Surgery Department, Royal Free Hospital, London, United Kingdom; Institute for Immunity and Transplantation, UCL, London, United Kingdom; Department of Cell and Developmental Biology, University College London, London, United Kingdom; Institute for Immunity and Transplantation, UCL, London, United Kingdom

**Keywords:** Cytomegalovirus, circadian rhythm, solid organ transplantation, monitoring, prophylaxis, preemptive

## Abstract

**Background:**

Cytomegalovirus (CMV) is an important opportunistic pathogen after transplantation. Some virological variation in transplant recipients is explained by donor and recipient CMV serostatus, but not all. Circadian variability of herpesviruses has been described, so we investigated the effect of time of day of transplantation on posttransplant CMV viremia.

**Methods:**

We performed a retrospective analysis of 1517 patients receiving liver or kidney allografts at a single center from 2002 to 2018. All patients were given preemptive therapy with CMV viremia monitoring after transplantation. Circulatory arrest and reperfusion time of donor organ were categorized into 4 periods. Patients were divided into serostatus groups based on previous CMV infection in donor and recipient. CMV viremia parameters were compared between time categories for each group. Factor analysis of mixed data was used to interrogate this complex data set.

**Results:**

Live-donor transplant recipients were less likely to develop viremia than recipients of deceased-donor organs (48% vs 61%; *P* < .001). After controlling for this, there was no evidence of time of day of transplantation affecting CMV parameters in any serostatus group, by logistic regression or factor analysis of mixed data.

**Discussion:**

We found no evidence for a circadian effect of transplantation on CMV viremia, but these novel results warrant confirmation by other centers.

## Teaser

Using a large data set on preemptive cytomegalovirus therapy for solid-organ transplant recipients, we found no association between time of day of transplantation and cytomegalovirus viremia but found increased viremia in recipients of deceased-donor compared with live-donor transplants.

Cytomegalovirus (CMV) is a member of the Herpesviridae family of double-stranded DNA viruses. It is an important opportunistic pathogen that affects a high proportion of patients after transplantation [1, 2]. Given its frequency and clinical severity, transplant centers currently use either of 2 protocols for controlling CMV viremia and preventing the high viral loads associated with end-organ disease. Patients may receive preemptive therapy, in which they are monitored for evidence of CMV DNA in blood and then treated once a viremia threshold is reached to prevent high viral loads, or they may be given antiviral prophylaxis from the time of transplantation onward [3, 4]. Both of these strategies are clinically effective, and both are recommended in current clinical guidelines [2]. Our center uses preemptive therapy, which allows us to detect and characterize patients with CMV infection. Several virological parameters have been defined—including the proportion of transplant recipients in whom viremia develops, the peak viral load, and the duration of viremia—that are robust enough to be used as the primary end points in clinical trials [3, 5, 6].

Variation in posttransplant virological parameters illustrates that transplant recipients have markedly different phenotypes. Some of this variation is explained by previous CMV infection in either the donor or the recipient of the organ, as measured by the presence of CMV immunoglobulin G (IgG) antibodies in the donor or recipient before transplantation, producing 3 subgroups of transplant recipients: donor positive for CMV IgG and recipient negative (D+R−), donor negative and recipient positive (D−R+), and both donor and recipient positive (D+R+) [3]. The D+R− subgroup carries the highest risk of viremia after transplantation. These patients have not previously been exposed to CMV, and thus viremia follows primary infection acquired from the new organ. These patients have significantly higher viral load parameters than both the D+R+ and D−R+ groups [3]. The D−R+ subgroup has the lowest risk of viremia and peak viral load. These patients have previously been infected with CMV, so viremia results from reactivation of their own latent CMV. Viremia in the D+R+ subgroup represents a mixture of reinfection and reactivation, and as such carries intermediate risk of viremia [3].

Subgrouping transplant recipients according to donor/recipient CMV serostatus does not completely explain posttransplant viral load variability. One factor that may affect CMV viremia is the timing of transplantation. Viruses are obligate intracellular pathogens that rely on host metabolism for their replication. There is extensive evidence from cell culture and animal models that virus replication and reactivation is modulated according to the time of day and that this effect can be blocked by deletion of genes that mediate cellular circadian rhythms (reviewed in [7]). This effect has been specifically shown in herpesviruses, including in animal models of CMV [8–11]. Circadian rhythms also affect immune function ([12]; reviewed in [13]), and there is evidence specifically relating to changing viral immunity based on diurnal rhythms [14]. Given this background, we believed it was important to determine whether circadian rhythms could explain some of the variation in phenotypes seen with CMV after transplantation. This could help further risk stratify patients in clinical and trial settings and could lead to new therapeutic options if drugs able to reset molecular clocks were developed.

## MATERIALS AND METHODS

### Patients

We carried out a retrospective case note analysis of transplant recipients who received liver or kidney allografts rom 2002 to 2018 at the Royal Free Hospital, London, United Kingdom. All patients were given preemptive therapy using whole-blood samples collected twice a week for the first 60 days after transplantation, then once a week until day 90. CMV load was monitored with quantitative polymerase chain reaction, using a method reported elsewhere [15], until September 2017; after that point, a commercial assay with the same viral load threshold of 200 copies/mL was used (Qiagen artus CMV QS-RGQ Kit).

Until 1 July 2012, preemptive therapy was started in all patients when the viral load was ≥3000 genomes per milliliter (≥2520 IU/mL). The protocol then changed for the D+R− subset, who were treated when their first polymerase chain reaction–positive result was obtained [16]. Patients were excluded from analysis if they received >1 transplant over the study period, if they received multiple organs in a single transplantation procedure, if their CMV serostatus was not recorded, if the transplantation was performed outside the Royal Free Hospital, or if the transplant documentation was not available.

We analyzed demographic data (recipient sex and age, donor sex and age, organ transplanted, and whether the donor was live or deceased). To capture potential circadian effects on the donor organ at time of removal or introduction into a new host, we recorded both the time of circulatory arrest of the donor (or in live-donor transplants the time of arterial clamping) and the reperfusion time of the organ in the recipient. These parameters were collected from patient notes and the times of day were categorized into 4 classifications before analysis: morning (4–10 am), day (10 am to 4 pm), evening (4–10 pm), and night (10 pm to 4 am). Virological data were collected as in previous studies and included presence of viremia, duration of viremia, peak viral load, and treatment requirement. The study was approved by the South Central–Oxford B Research Ethics Committee.

### Statistical Analysis

Data were analyzed separately for the 3 distinct subgroups of patients (D+R−, D−R+, and D+R+). Statistical analysis of the baseline data and viremic parameters was carried out using Stata 15 software, with χ^2^ and Kruskal-Wallis statistical tests used to provide *P* values [17]. Logistic regression was used to examine the associations of reperfusion time and circulatory arrest time with the outcome of presence of viremia. Again, analyses were stratified by donor and recipient status. Multivariable analyses were adjusted for factors posited as confounders for the associations: namely organ type, live or deceased donor, sex of donor, sex of recipient, age of donor, age of recipient, and calendar season. Visual representations of the virological parameters for each time of day were created in Microsoft Excel.

### Factor Analysis of Mixed Data

Because the data set contained a mixture of qualitative and quantitative variables, we used factor analysis of mixed data (FAMD), an approach similar to principal component analysis that is suitable for data sets containing mixed variable types. FAMD was performed on data from patients, using R software (version 4.1.0), RStudio (version 1.4.1717), and R packages FactoMineR [18] and factoextra.

## RESULTS

A total of 1517 patients were included in the analysis: 243 in the D+R−, 543 in the D+R+, and 731 in the D+R+ subgroup.

### Time of Day

#### Baseline Characteristics

Demographic characteristics of transplant recipients, divided by the time of circulatory arrest in the donor and of reperfusion in the recipient, are shown in Table 1. There was no evidence of an association between recipient sex and either circulatory arrest time (*P* = .56) or reperfusion time (*P* = .50). There was also no evidence for an association between donor sex and circulatory arrest time (*P* = .11), but there was a statistically significant difference in reperfusion time, with relatively fewer men with reperfusion times during the day (49% vs 57% for morning and evening and 59% for night; *P* = .006).

**Table 1.** Demographic Data for Patients by Time of Circulatory Arrest or Reperfusion

Demographic Variable	Patients, No. (%)				*P* Value^a^
	Morning	Day	Evening	Night	
Recipient sex					
CA time					
Female	177 (37)	177 (35)	69 (40)	130 (36)	.56
Male	297 (63)	334 (65)	102 (60)	231 (64)	
RP time					
Female	67 (38)	247 (38)	154 (37)	85 (32)	.51
Male	110 (62)	410 (62)	267 (63)	177 (68)	
Donor sex					
CA time					
Female	202 (43)	253 (50)	72 (42)	171 (47)	.11
Male	272 (57)	258 (50)	99 (58)	190 (53)	
RP time					
Female	76 (43)	336 (51)	179 (43)	107 (41)	<.01
Male	101 (57)	321 (49)	242 (57)	155 (59)	
Donor state					
CA time					
Deceased	473 (100)	276 (54)	169 (99)	361 (100)	<.001
Live	1 (0)	235 (46)	2 (1)	0 (0)	
RP time					
Deceased	177 (100)	471 (72)	370 (89)	261 (100)	<.001
Live	0 (0)	186 (28)	51 (12)	1 (0)	
Organ					
CA time					
Kidney	191 (40)	351 (69)	88 (51)	167 (46)	<.001
Liver	283 (60)	160 (31)	83 (49)	194 (54)	
RP time					<.001
Kidney	124 (70)	306 (47)	204 (48)	163 (62)	
Liver	53 (30)	351 (53)	217 (52)	99 (38)	

Abbreviations: CA, circulatory arrest; RP, reperfusion.

*P* values generated using the Kruskal-Wallis test, comparing the 4 time periods: morning (4–10 am), day (10 am to 4 pm), evening (4–10 pm), and night (10 pm to 4 am).

As expected, there was evidence of a difference in time of day of transplantation between deceased-donor and live-donor transplants, with organs from live donors more frequently transplanted during in the day (46% of daytime circulatory arrest times were in live-donor transplants vs 0%–1% in other time periods, and 28% of daytime reperfusion times were in live-donor transplants vs 12% in the evening and 0% in the night and morning; both *P* < .001). We also found a difference in time of day for kidney versus liver transplantation, with relatively more kidney circulatory arrest times occurring during the day and reperfusion times at night or in the morning (circulatory arrest times for kidney, 40% morning, 69% day, 51% evening, and 46% night; reperfusion time for kidney, 70%, 47%, 48%, and 62%, respectively; both *P* < .001).

Supplementary Table 1 shows the effect of donor state (deceased or live) and the organ transplanted (kidney or liver) on the likelihood of CMV viremia in the recipient. There was a significantly higher risk of viremia with deceased-donor compared with live-donor transplants; viremia developed in 61% of deceased-donor versus 48% of live-donor transplant recipients ( *P* < .001).

#### D+R− Subgroup

**Figure 1.**
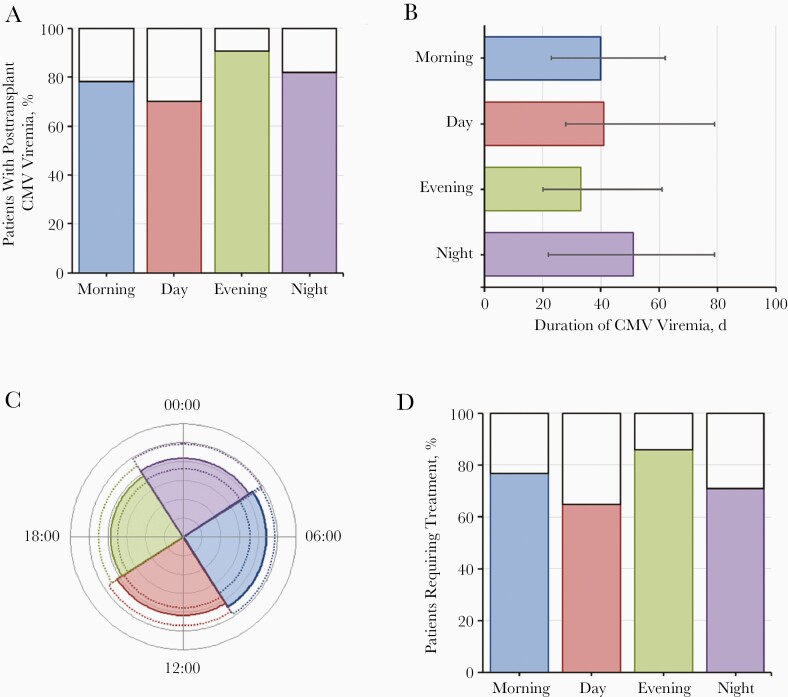
Graphic representation of the effect of circulatory arrest time on cytomegalovirus (CMV) parameters in the cohort with transplant donors positive and recipients negative for CMV immunoglobulin G (D+R−). *A,* Percentage of patients with posttransplant CMV viremia. *B,* Median duration of CMV viremia (with interquartile range [IQR]). *C,* Median peak viral load (*colored segments*) with IQR (*dotted lines*). *D,* Percentage of patients requiring treatment. All parameters are shown by time of circulatory arrest.

**Figure 2.**
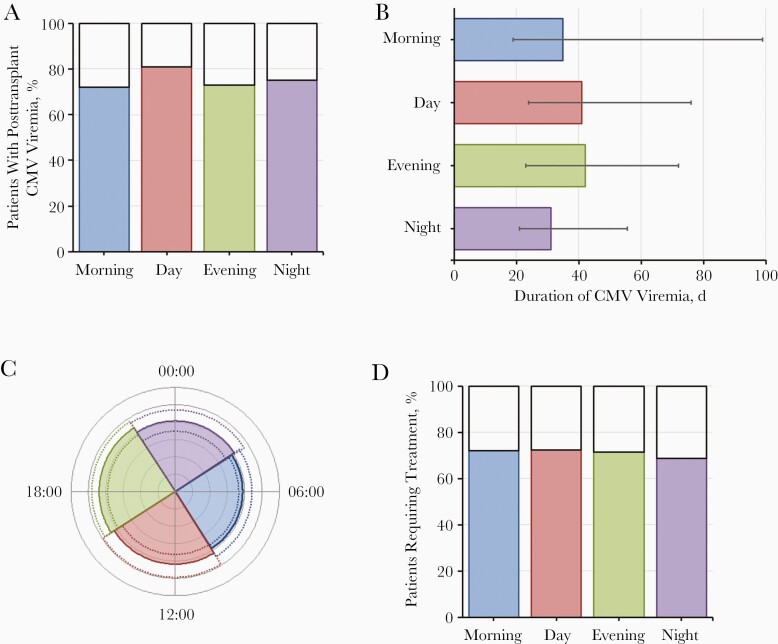
Graphic representation of the effect of reperfusion time on cytomegalovirus (CMV) parameters in the cohort with transplant donors positive and recipients negative for CMV immunoglobulin G (D+R−). *A,* Percentage of patients with posttransplant CMV viremia. *B,* Median duration of CMV viremia (with interquartile range [IQR]). *C,* Median peak viral load (*colored segments*) with IQR (*dotted lines*). *D,* Percentage of patients requiring treatment. All parameters are shown by time of reperfusion.

Logistic regression analysis found no evidence of an association between viremia and either circulatory arrest time or reperfusion time in the D+R− subgroup (see Table 2). The same result was found when analysis was limited to deceased-donor patients only (see Supplementary Table 5). There was also no evidence of different viremic parameters resulting from differences in circulatory arrest or reperfusion times (Supplementary Table 2). Figures 1 and 2 are graphic representations of the effect of circulatory arrest (Figure 1) and reperfusion (Figure 2) times on CMV parameters in the D+R− cohort.

**Table 2.** Association Between Time of Reperfusion and Circulatory Arrest With Presence of Cytomegalovirus Viremia, According to Donor and Recipient Status

Time of RP and CA by Cohort	Unadjusted^a^			Adjusted^a,b^		
	OR	95% CI	*P* Value	OR	95% CI	*P* Value
D+R− (n = 243)						
RP time						
Morning	.60	.22–1.62	.54	.69	.21–2.26	.43
Day	1.00	…		1.00	…	
Evening	.63	.33–1.27		.51	.22–1.15	
Night	.70	.28–1.77		.62	.20–1.95	
CA time						
Morning	1.51	.74–3.07	.13	.89	.32–2.44	.44
Day	1.00	…		1.00	…	
Evening	4.03	.88–18.5		2.70	.46–15.98	
Night	1.91	.84–4.31		1.56	.51–4.75	
D+R+ (n = 731)						
RP time	2.06	1.22–3.46		1.48	.83–2.63	
Morning			.01			.58
Day	1.00	…		1.00	…	
Evening	1.32	.92–1.90		1.16	.78–1.72	
Night	1.65	1.08–2.53		1.25	.77–2.03	
CA time						
Morning	1.49	1.02–2.16	.01	.98	.61–1.57	.70
Day	1.00	…		1.00	…	
Evening	1.98	1.16–3.37		1.36	.74–2.49	
Night	1.68	1.13–2.48		1.01	.62–1.64	
D−R+ (n = 543)						
RP time						
Morning	1.91	1.09–3.34	.09	1.72	.95–3.17	.21
Day	1.00	…		1.00	…	
Evening	1.43	.95–2.15		1.45	.94–2.22	
Night	1.39	.86–2.25		1.24	.74–2.10	
CA time						
Morning	1.12	.73–1.71	.44	1.33	.79–2.24	.49
Day	1.00	…		1.00	…	
Evening	1.61	.73–1.71		1.65	.86–3.17	
Night	1.19	.74–1.92		1.29	.74–2.26	

Abbreviations: CA, circulatory arrest; CI, confidence interval; D+R−, transplant donor cytomegalovirus (CMV) positive and recipient CMV negative; D+R+, donor and recipient both CMV positive; D−R+, donor CMV negative and recipient CMV positive; OR, odds ratio; RP, reperfusion.

Results from logistic regression models.

Adjusted for organ type, live or deceased donor, sex of donor and recipient, age of donor and recipient, and calendar season.

#### D−R+ Subgroup

**Figure 3.**
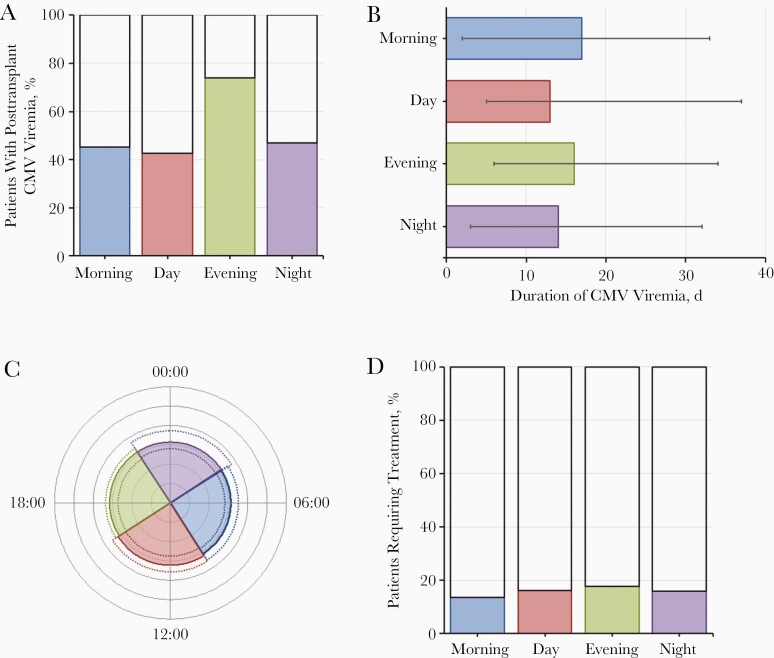
Graphic representation of the effect of circulatory arrest time on cytomegalovirus (CMV) parameters in the cohort with transplant donors negative and recipients positive for CMV immunoglobulin G (D−R+). *A,* Percentage of patients with posttransplant CMV viremia. *B,* Median duration of CMV viremia (with interquartile range [IQR]). *C,* Median peak viral load (*colored segments*) with IQR (*dotted lines*). *D,* Percentage of patients requiring treatment. All parameters are shown by time of circulatory arrest.

**Figure 4.**
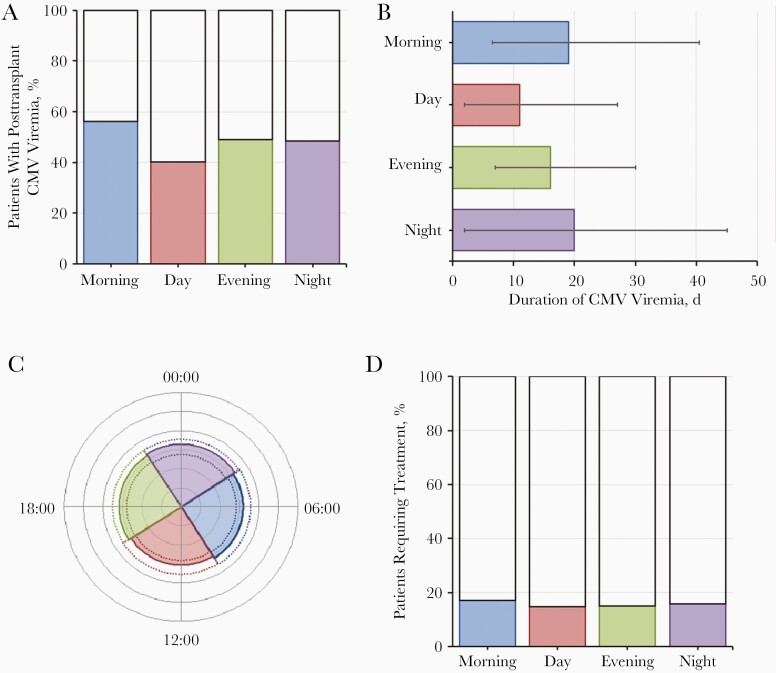
Graphic representation of the effect of reperfusion time on cytomegalovirus (CMV) parameters in the cohort with transplant donors negative and recipients positive for CMV immunoglobulin G (D−R+). *A,* Percentage of patients with posttransplant CMV viremia. *B,* Median duration of CMV viremia (with interquartile range [IQR]). *C,* Median peak viral load (*colored segments*) with IQR (*dotted lines*). *D,* Percentage of patients requiring treatment. All parameters are shown by time of reperfusion.

Logistic regression analysis also found no evidence of an association between viremia and circulatory arrest or reperfusion times in the D−R+ subgroup (see Table 2). The same result was found when analysis was limited to deceased-donor transplant recipients (see Supplementary Table 5). There was also no evidence of different viremic parameters for circulatory arrest or reperfusion times (Supplementary Table 3). Figures 3 and 4 are graphic representations of the effects of circulatory arrest (Figure 3) and reperfusion (Figure 4) times on CMV parameters in the D−R+ cohort.

#### D+R+ Subgroup

As in the other subgroups, logistic regression analysis found no evidence of an association between viremia and either circulatory arrest or reperfusion times in the D+R+ subgroup (see Table 2). The same result was found when analysis was limited to deceased-donor transplant recipients (see Supplementary Table 5). There was also no evidence of different viremic parameters for circulatory arrest or reperfusion time (Supplementary Table 4).

### Seasons

Results for the effect of season on CMV viremia for each serogroup are shown in Supplementary Table 6. There was no evidence for an association with season of transplantation and development of viremia in any serotype subgroup. There was also no seasonal association with any viremic parameter in the serotype subgroups, except for a shorter duration of viremia in winter for D+R+ patients (15 days in winter vs 18.5, 21, and 27 days in spring, summer, and autumn, respectively; *P* = .02), however, no consistent pattern was seen in other virological parameters for this season/serotype group.

### Multivariate Analysis

Given the large numbers of variables present within the data set, we perform a multivariate analysis to investigate whether any particular factors were associated with CMV viremia when considered as a whole. Furthermore, given that live transplants tended to be skewed toward a particular time of day, we limited the FAMD analysis to only deceased-donor transplants.

**Figure 5.**
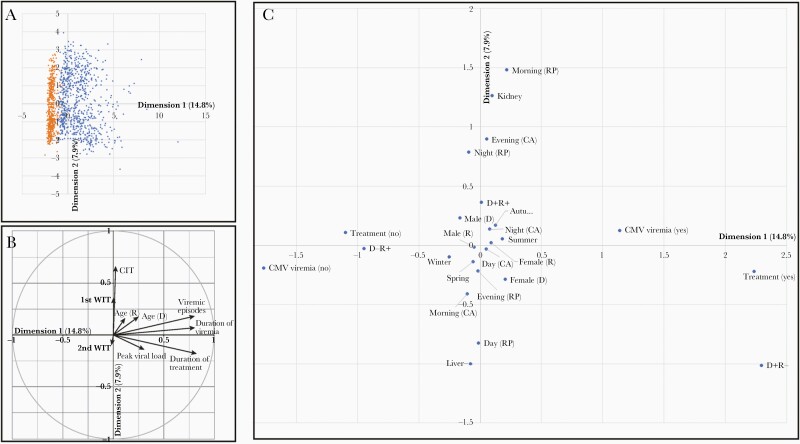
Factor analysis of mixed data reveals no strong impact of time of day or season on likelihood of development of cytomegalovirus (CMV) viremia. *A,* Plot of individual patients on the first 2 dimensions arising from factor analysis of mixed data (FAMD), colored according to development of viremia (*orange* [no viremia detected] or *light blue* [developed viremia]). Parenthetical numbers represent percentage of variation explained by the associated dimension. *B,* Weightings of quantitative variables for the first 2 FAMD dimensions. Abbreviations: CIT, cold ischemia time; D, transplant donor; R, transplant recipient; WIT, warm ischemia time. *C,* Weightings of categorical variables for the first 2 FAMD dimensions. Where required, labels reflect both the variable being categorized and the specific category. Abbreviations: CA, circulatory arrest; D−R+, donor CMV negative and recipient CMV positive; D+R+, donor and recipient both positive; D+R−, donor positive and recipient negative; RP, reperfusion.

Figure 5A displays the relative positions for each patient on the first 2 dimensions resulting from FAMD. Patients in whom viremia developed cluster distinctly from those without viremia, with the variation between the 2 groups driven strongly by the first dimension 1 (14.8%). As can be seen in Figure 5B, which represents the weighting of the quantitative variables for the first 2 dimensions, the first dimension is driven strongly by CMV viremia–related factors (duration of treatment, duration of viremia, number of viremic episodes, and peak viremia), so it is unsurprising that the 2 patient populations diverge based on these criteria. The only other factor that is appreciably weighted in this direction is the age of the donor, which is in fact weighted almost as heavily as peak viral load, although these are both weighted relatively lightly compared with the other viremia-related variables. Meanwhile, dimension 2 (7.9%) is most strongly influenced by the cold ischemia time.

When evaluating the impact of the qualitative variables (Figure 5C), we again observe that factors associated with CMV viremia are weighted heavily along the first dimension, with both confirmed CMV viremia and requirement for CMV treatment having a strong positive weighting, while the lack of CMV viremia and the lack of treatment have strong negative weightings. We also observe that D+R− transplants are also strongly associated with the positive direction of dimension 1, while D−R+ transplants are strongly associated with the negative direction, with D+R+ transplants in between. This recapitulates the well-characterized stratification of risk for CMV viremia with solid organ transplantations, with D+R− recipients at highest risk of CMV viremia, D+R+ recipients less so, and D−R+ recipients at the least relative risk. It also confirms that dimension 1 effectively represents “risk of CMV viremia,” and thus factors weighted along dimension 1 likely have an impact on the risk of CMV viremia after liver transplantation. The factors that drive dimension 1 also have extremely small weightings along dimension 2, indicating that dimension 2 is not associated with the likelihood of CMV viremia.

While the weightings of the other qualitative variables along the first dimension are relatively small compared with the variables discussed above, there are some interesting points to note. Namely, having a female donor is weighted approximately 0.25 in the positive direction, while having a male donor is weighted approximately 0.25 in the negative direction, suggesting that having a female donor leads to a greater risk of CMV viremia. This may be partially explained by a higher frequency of D+ organs coming from female compared with male donors (226 of 350 [64.6%] vs 203 of 370 [54.9%], respectively). In terms of the impact of seasons, winter appears to be the least “risky” season for CMV viremia associated with transplantation, although the impact of seasonality is minor at most and may be reflect a wide range of factors. Finally, all variables associated with “time of day” have extremely small weightings along the first dimension, indicating that they have little impact on the risk of CMV viremia.

## DISCUSSION

We did not find any evidence of an association between time of day of solid organ transplantation and later viremia in any of the donor-recipient subgroups. There were also no seasonality differences in our data. These findings were confirmed with use of the FAMD, showing little effect of timing parameters.

To the best of our knowledge, ours is the first study to address this question, so we suggest that colleagues in other transplant centers examine their data to facilitate a future meta-analysis. Our center uses preemptive therapy, and it would be interesting to see if those using prophylaxis have evidence of circadian effects linked to late-onset disease and the development of antiviral resistance [4, 19]. Future analyses would be facilitated by the creation of novel statistical methods to examine data that might have circular, cycling relationships across a 24-hour period. Our data set did not include time of day of CMV DNA samples, but a future study could examine this relationship, looking for circadian associations.

Our study has several possible limitations. First, there are multiple possible confounders masking an association. For example, we do not know the occupation of donors or recipients and whether they were shift workers, and we do not have information about preceding hospital admission, as the hospital environment is known to disrupt circadian rhythms. Moreover many patients will be admitted to an intensive care unit after transplantation, with well-documented circadian disruption (reviewed in [20]). All of these patients will have received immunosuppression after transplantation, some including glucocorticoids, which are well known to affect the circadian rhythm of the immune system [21]. These factors are impossible to control for, as—importantly in this study—the population is representative of the standard population of solid organ transplant recipients.

This study did confirm previous study findings. First, the serotype subgroups showed a risk pattern similar to that inn previous studies, with the D+R− subgroup the most likely to develop viremia, and the D−R+ subgroup the least likely [3]. Second, we found an increased risk of viremia in patients who received a deceased-donor rather than a live-donor transplant, as reported for a 2021 study in which patients received antiviral prophylaxis [22]. Confirming this association in a center using preemptive rather than prophylactic therapy.

As expected, we did find deceased-donor transplant recipients had longer median cold ischemia times (613 vs 158 minutes in live-donor transplant recipients), and we hypothesized that this could be the cause of increased CMV viremia in these patients; however, there was no evidence that a longer cold ischemia time was associated with viremia in either simple analysis or the FAMD. An alternative explanation for this finding could be that CMV replicates faster in a deceased-donor organ before reperfusion owing to decreased tissue perfusion, or that the immune response in the recipient of a deceased-donor organ favors CMV reactivation. High-dose corticosteroids are used as “conditioning” for deceased-donor organ recipients in some settings and could explain CMV reactivation in these organs; however this regimen is not routinely used in UK hospitals. We suggest that future randomized controlled trials of antiviral drugs or vaccines against CMV should consider stratifying by type of donor organ.

In conclusion, the current study did not find any circadian or seasonal effect on the development of CMV viremia after transplantation. However, this is the first study of its kind, and we would therefore encourage a similar analysis at other transplant centers, particularly comparing centers using prophylactic CMV regimens. We did confirm the finding that deceased-donor transplant recipients are at higher risk of CMV viremia than live-donor recipients, extending this observation to patients receiving preemptive therapy, and we therefore suggest adjustment for or stratification of these patients in future studies.

## Supplementary Data

Supplementary materials are available at *The Journal of Infectious Diseases* online. Supplementary materials consist of data provided by the author that are published to benefit the reader. The posted materials are not copyedited. The contents of all supplementary data are the sole responsibility of the authors. Questions or messages regarding errors should be addressed to the author.

jiac055_suppl_Supplementary_Tables

## Notes

Presented in part: IDWeek 2018, San Francisco, California, 3–7 October 2018.

***Acknowledgments***. We thank Helene Borrmann for her critical appraisal of the final manuscript and her expertise in the circadian regulation of viral infections.

***Financial support.*** This work was supported by the Wellcome Trust (collaborative grant 204870) and the Medical Research Council (grant MR/RO21384/1 to M. R.).

***Potential conflicts of interest.*** All authors: No reported conflicts. All authors have submitted the ICMJE Form for Disclosure of Potential Conflicts of Interest. Conflicts that the editors consider relevant to the content of the manuscript have been disclosed.
