# Supplementary material for: Are the Patterns of Cytomegalovirus Viral Load Seen After Solid Organ Transplantation Affected by Circadian Rhythm?
Source: J Infect Dis. 2022 Feb 20;226(2):357–65. doi: 10.1093/infdis/jiac055 (PMC9400432; doi:10.1093/infdis/jiac055)
Supplement: jiac055_suppl_Supplementary_Tables [file jiac055_suppl_supplementary_tables.docx]

Supplementary tables

*Supplementary table 1:*  Relationship between donor state (cadaveric/live) and the organ transplanted (kidney/liver) on the likelihood of the recipient developing a CMV viremia.

|  | Donor state | | | Organ transplanted | | |
| --- | --- | --- | --- | --- | --- | --- |
|  | Cadaveric | Live | P value | Kidney | Liver | P value |
| Viremia? N (%) | | | | | | |
| No | 501 (39%) | 123 (52%) | <0.001 | 333 (42%) | 291 (40%) | 0.590 |
| Yes | 778 (61%) | 115 (48%) |  | 464 (58%) | 429 (60%) |  |
| Duration viremia (days) | | | | | | |
| Median | 23 | 27 | 0.300 | 27 | 20 | 0.178 |
| IQR | 8-42 | 7-55 |  | 8-51 | 7-36 |  |
| Peak (copies/ml) | | | | | | |
| Median | 3064 | 2800 | <0.001 | 2582.5 | 3371 | 0.111 |
| IQR | 907-9468 | 641-11135 |  | 828.5-7090.5 | 961-12048 |  |
| Treated? N (%) | | | | | | |
| No | 857 (67%) | 178 (75%) | 0.018 | 568 (71%) | 467 (65%) | 0.007 |
| Yes | 422 (33%) | 60 (25%) |  | 229 (29%) | 253 (35%) |  |

*Supplementary Table 2 The effect of transplantation timing on CMV parameters in D+R- patients*

|  |  | Morning | Day | Evening | Night | P value |
| --- | --- | --- | --- | --- | --- | --- |
| Viremia? N (%) | | | | | | |
| Circulatory arrest time | No | 16 (22%) | 28 (30%) | 2 (10%) | 10 (18% | 0.147 |
|  | Yes | 57 (78%) | 66 (70%) | 19 (90%) | 45 (82%) |  |
| Reperfusion time | No | 7 (28%) | 22 (19%) | 19 (27%) | 8 (25%) | 0.539 |
|  | Yes | 18 (72%) | 94 (81%) | 51 (73%) | 24 (75%) |  |
| Duration viremia (days) | | | | | | |
| Circulatory arrest time | Median | 40 | 41 | 33 | 51 | 0.657 |
|  | IQR | 23-62 | 28-79 | 20-61 | 22-79 |  |
| Reperfusion time | Median | 35 | 41 | 42 | 31 | 0.730 |
|  | IQR | 19-99 | 24-76 | 23-72 | 21-55.5 |  |
| Peak viral load (copies/ml) | | | | | | |
| Circulatory arrest time | Median | 25692 | 14726 | 7202 | 14870 | 0.457 |
|  | IQR | 3510-73291 | 5747-52072 | 3111-31478 | 4074-85691 |  |
| Reperfusion time | Median | 7666.5 | 15024 | 24000 | 11791.5 | 0.143 |
|  | IQR | 4606-26131 | 4161-85691 | 7793-63884 | 3005.5-50658.5 |  |
| Treated? N (%) | | | | | | |
| Circulatory arrest time | No | 17 (23%) | 33 (35%) | 3 (14%) | 16 (29%) | 0.165 |
|  | Yes | 56 (77%) | 61 (65%) | 18 (86%) | 39 (71%) |  |
| Reperfusion time | No | 7 (28%) | 32 (28%) | 20 (29%) | 10 (31%) | 0.982 |
|  | Yes | 18 (72%) | 84 (72%) | 50 (71%) | 22 (69%) |  |

*Supplementary Table 3 The effect of transplantation timing on CMV parameters in D-R+ patients.*

|  |  | Morning | Day | Evening | Night | P value |
| --- | --- | --- | --- | --- | --- | --- |
| Viremia? N (%) | | | | | | |
| Circulatory arrest time | No | 110 (55%) | 85 (57%) | 31 (46%) | 67 (53%) | 0.438 |
|  | Yes | 91 (45%) | 63 (43%) | 37 (54%) | 59 (47%) |  |
| Reperfusion time | No | 28 (44%) | 138 (60%) | 78 (51%) | 49 (52%) | 0.087 |
|  | Yes | 36 (56%) | 93 (40%) | 75 (49%) | 46 (48%) |  |
| Duration viremia (days) | | | | | | |
| Circulatory arrest time | Median | 17 | 13 | 16 | 14 | 0.457 |
|  | IQR | 2-33 | 5-37 | 6-34 | 3-32 |  |
| Reperfusion time | Median | 19 | 11 | 16 | 20 | 0.135 |
|  | IQR | 6.5-40.5 | 2-27 | 7-30 | 2-45 |  |
| Peak viral load (copies/ml) | | | | | | |
| Circulatory arrest time | Median | 1313 | 1676 | 1400 | 1377 | 0.615 |
|  | IQR | 648-3254 | 566-3641 | 508-2254 | 630-5287 |  |
| Reperfusion time | Median | 1514.5 | 1127 | 1551 | 1934 | 0.829 |
|  | IQR | 678.5-3673.5 | 633-3371 | 634-3313 | 541-3412 |  |
| Treated? N (%) | | | | | | |
| Circulatory arrest time | No | 174 (87%) | 124 (84%) | 56 (82%) | 106 (84%) | 0.811 |
|  | Yes | 27 (13%) | 24 (16%) | 12 (18%) | 20 (16%) |  |
| Reperfusion time | No | 53 (83%) | 197 (85%) | 130 (85%) | 80 (84%) | 0.967 |
|  | Yes | 11 (17%) | 34 (15%) | 23 (15%) | 15 (16%) |  |

*Supplementary Table 4 The effect of transplantation timing on CMV parameters in D+R+ patients.*

|  |  | Morning | Day | Evening | Night | P value |
| --- | --- | --- | --- | --- | --- | --- |
| Viremia? N (%) | | | | | | |
| Circulatory arrest time | No | 71 (35.5) | 121 (45.0) | 24 (29.3) | 59 (32.8) |  |
|  | Yes | 129 (64.5) | 148 (55.0) | 58 (70.7) | 121 (67.2) | 0.013 |
| Reperfusion time | No | 24 (27.3) | 135 (43.6) | 73 (36.9) | 43 (31.9) |  |
|  | Yes | 64 (72.7) | 175 (56.5) | 125 (63.1) | 92 (68.2) | 0.014 |
| Duration viremia (days) | | | | | | |
| Circulatory arrest time | *Median* | 24 | 22.5 | 15.5 | 20 |  |
|  | IQR | 9-45 | 5.5-39.5 | 1-33 | 6-37 | 0.065 |
| Reperfusion time | Median | 23.5 | 21 | 21 | 22 |  |
|  | IQR | 11.5-41.5 | 8-38 | 3-40 | 3-40 | 0.065 |
| Peak viral load (copies/ml) | | | | | | |
| Circulatory arrest time | Median | 3160 | 2706.5 | 1839 | 3025 |  |
|  | IQR | 658-6868 | 715.5-7248 | 855-4824 | 921-6026 | 0.079 |
| Reperfusion time | Median | 2851 | 3046 | 2556 | 7361 |  |
|  | IQR | 874.5-6522 | 840-6816 | 713-6008 | 783-7361 | 0.062 |
| Treated? N (%) | | | | | | |
| Circulatory arrest time | No | 131 (65.5) | 198 (73.6) | 120 (66.7) | 57 (69.5) |  |
|  | Yes | 69 (34.5) | 71 (26.4) | 60 (33.3) | 25 (30.5) | 0.233 |
| Reperfusion time | No | 56 (63.6) | 220 (71.0) | 139 (70.2) | 91 (67.4) |  |
|  | Yes | 32 (36.4) | 90 (29.0) | 59 (29.8) | 44 (32.6) | 0.566 |

Supplementary Table 5 Association between time of reperfusion and circulatory arrest with presence of viremia, according to donor/recipient status restricted to cadaveric donors

|  |  | **Unadjusted** | | | **Adjusted*** | | |
| --- | --- | --- | --- | --- | --- | --- | --- |
|  |  | **OR** | **95% CI** | **P** | **OR** | **95% CI** | **P** |
| **D+R- (n=200)** |  |  |  |  |  |  |  |
| Reperfusion time | Morning | 0.31 | 0.10, 0.94 | 0.071 | 0.62 | 0.18, 2.19 | 0.27 |
|  | Day | 1.00 | - |  | 1.00 | - |  |
|  | Evening | 0.36 | 0.15, 0.89 |  | 0.37 | 0.14, 1.00 |  |
|  | Night | 0.41 | 0.14, 1.22 |  | 0.62 | 0.17, 2.21 |  |
| Circulatory arrest time | Morning | 0.87 | 0.36, 2.11 | 0.13 | 0.83 | 0.30, 2.31 | 0.41 |
|  | Day | 1.00 | - |  | 1.00 | - |  |
|  | Evening | 2.31 | 0.46, 11.6 |  | 2.82 | 0.47, 17.0 |  |
|  | Night | 1.10 | 0.41, 2.90 |  | 1.45 | 0.47, 4.54 |  |
| **D+R+ (n=589)** |  |  |  |  |  |  |  |
| Reperfusion time | Morning | 1.53 | 0.88, 2.65 | 0.45 | 1.43 | 0.79, 2.58 | 0.68 |
|  | Day | 1.00 | - |  | 1.00 | - |  |
|  | Evening | 1.06 | 0.69, 1.63 |  | 1.11 | 0.71, 1.74 |  |
|  | Night | 1.23 | 0.77, 1.95 |  | 1.22 | 0.73, 2.01 |  |
| Circulatory arrest time | Morning | 1.01 | 0.64, 1.60 | 0.72 | 0.99 | 0.62, 1.60 | 0.72 |
|  | Day | 1.00 | - |  | 1.00 | - |  |
|  | Evening | 1.36 | 0.74, 2.48 |  | 1.36 | 0.73, 2.54 |  |
|  | Night | 1.12 | 0.70, 1.81 |  | 1.00 | 0.61, 1.63 |  |
| **D-R+ (n=490)** |  |  |  |  |  |  |  |
| Reperfusion time | Morning | 1.88 | 1.06, 3.33 | 0.16 | 1.57 | 0.85, 2.88 | 0.51 |
|  | Day | 1.00 | - |  | 1.00 | - |  |
|  | Evening | 1.28 | 0.83, 1.99 |  | 1.24 | 0.78, 1.95 |  |
|  | Night | 1.37 | 0.84, 2.25 |  | 1.15 | 0.68, 1.95 |  |
| Circulatory arrest time | Morning | 1.24 | 0.76, 2.04 | 0.33 | 1.34 | 0.80, 2.25 | 0.46 |
|  | Day | 1.00 | - |  | 1.00 | - |  |
|  | Evening | 1.79 | 0.95, 3.36 |  | 1.69 | 0.88, 3.23 |  |
|  | Night | 1.32 | 0.77, 2.27 |  | 1.30 | 0.74, 2.27 |  |

Results from logistic regression models

*Adjusted for organ type, live/cadaveric donor, sex of donor, sex of recipient, age of recipient, age of donor and calendar season

D=Donor R=Recipient; OR=odds ratio; CI=confidence interval

*Supplementary table 6* Viremic variables in patients categorized into seasons for each donor/recipient serogroup

|  |  | Autumn | Spring | Summer | Winter | P value |
| --- | --- | --- | --- | --- | --- | --- |
| D+R- | | | | | | |
| Viremia?  N (%) | No | 11 (19%) | 13 (23%) | 19 (28%) | 13 (21%) | 0.667 |
|  | Yes | 47 (81%) | 43 (77%) | 49 (72%) | 48 (79%) |  |
| Duration viremia (days) | Median | 53 | 35 | 35 | 40 | 0.152 |
|  | IQR | 30-95 | 21-68 | 23-61 | 22.5-62.5 |  |
| Peak viral load (copies/ml) | Median | 16437 | 18172 | 8131 | 24209 | 0.107 |
|  | IQR | 8955-98328 | 2830-53019 | 4074-31723 | 5425-75371 |  |
| Treated?  N (%) | No | 14 (24%) | 16 (29%) | 23 (34%) | 16 (26%) | 0.649 |
|  | Yes | 44 (76%) | 40 (71%) | 45 (66%) | 45 (74%) |  |
| D-R+ | | | | | | |
| Viremia?  N (%) | No | 59 (46%) | 77 (56%) | 74 (55%) | 83 (59%) | 0.164 |
|  | Yes | 70 (54%) | 61 (44%) | 61 (45%) | 58 (41%) |  |
| Duration viremia (days) | Median | 14 | 16 | 24 | 13 | 0.141 |
|  | IQR | 4-29 | 4-30 | 7-42 | 2-23 |  |
| Peak viral load (copies/ml) | Median | 1175 | 1558 | 2071 | 1151 | 0.240 |
|  | IQR | 539-3254 | 703-4673 | 690-3953 | 633-2736 |  |
| Treated?  N (%) | No | 105 (81%) | 117 (85%) | 111 (82%) | 127 (90%) | 0.182 |
|  | Yes | 24 (19%) | 21 (15%) | 24 (18%) | 14 (10%) |  |

*Supplementary table 6 continued*

|  |  | Autumn | Spring | Summer | Winter | P value |
| --- | --- | --- | --- | --- | --- | --- |
| D+R+ | | | | | | |
| Viremia?  N (%) | No | 81 (41%) | 65 (37%) | 61 (34%) | 68 (39%) | 0.514 |
|  | Yes | 118 (59%) | 113 (63%) | 120 (66%) | 105 (61%) |  |
| Duration viremia (days) | Median | 18.5 | 21 | 27 | 15 | 0.015 |
|  | IQR | 2-38 | 7-39 | 11-43 | 3-33 |  |
| Peak viral load (copies/ml) | Median | 3095 | 2446 | 3174 | 2115 | 0.752 |
|  | IQR | 873-67975 | 1003-6051 | 641-9446 | 738-5700 |  |
| Treated?  N (%) | No | 57 (48%) | 61 (54%) | 55 (46%) | 58 (55%) | 0.430 |
|  | Yes | 61 (52%) | 52 (46%) | 65 (54%) | 47 (45%) |  |

*%, median and IQR data rounded to nearest whole number
